# Computational Evaluation of Novel PARP-1 Inhibitors for Breast Cancer: Docking, Molecular Dynamics, MM/GBSA, DFT and ADMET Calculations

**DOI:** 10.3390/ph19060914

**Published:** 2026-06-10

**Authors:** Charmy Twala, Penny Govender, Ephraim Marondedze, Krishna Govender

**Affiliations:** 1 Department of Chemical Sciences, Faculty of Science, University of Johannesburg, Doornfontein Campus, Johannesburg 2094, South Africa; 201106742@student.uj.ac.za (C.T.); pennyg@uj.ac.za (P.G.); ephraimm18@gmail.com (E.M.); 2 National Institute for Theoretical and Computational Sciences (NITheCS), Stellenbosch 7600, South Africa

**Keywords:** PARP1 Inhibition, computational chemistry, breast cancer, *BRCA* mutations, synthetic lethality, MD simulations, MM/GBSA, DFT

## Abstract

**Background/Objectives**: Poly (ADP-ribose) polymerase (PARP1) has emerged as a promising therapeutic target in human breast cancer particularly in *BRCA1/2* mutation carriers where a synthetic lethal interaction leads to massive tumor cell death upon specific inhibitors’ administration. Current clinically approved PARP inhibitors (Talazoparib and Olaparib) show outstanding therapeutic capabilities but suffer from severe side effects. Most importantly, some of them can cause life-threatening cardiotoxicity through hERG off-target effects. Here, we performed an extensive study to identify lead compounds with improved binding modes and favorable predicted pharmacokinetics using an integrated computational strategy. **Methods**: An artificial intelligence-driven drug design (AIDDISON™ v2023) workflow was employed to search ultra-large chemical space libraries for active compounds, which were then optimized via computer-aided methods to form a PARP-Tailored Database (PTD). This database was then analyzed through a virtual screening workflow, molecular docking studies, molecular dynamics (MD) simulations, MM/GBSA binding free energy calculations, DFT analysis and ADME/Tox predictions using the Schrödinger suite (v2023-2), MobaXterm v25.2, Gaussian 16.0, ProTox-3 and Pred-hERG v5.0 respectively. **Results**: Three compounds (1a–1c) were identified as promising candidates. Among them 1a appeared to be the most active compound with a favorable docking score (−9.488 kcal/mol) that is not only higher than 1b and 1c but also higher than that of Talazoparib (−6.778 kcal/mol). MD simulations of 1a–1c in the active site revealed an average RMSD of ~2.5–3.6 Å which is better compared to the parent Talazoparib (5.6 Å). Interestingly, on the 250 ns extended MD study, 1a exhibited a slightly reduced RMSD between 2.4 and 3.2 Å, whereas Talazoparib retained higher fluctuations of ~5 Å to 6 Å. MM/GBSA binding energy analysis indicated 1a to have better predicted binding affinity (−67.820 kcal/mol), which is also better than Talazoparib (−63.734 kcal/mol). DFT calculations showed good electronic properties and in silico ADMET studies also indicated 1a to have good drug-likeness and lower predicted hepatotoxicity and cardiotoxicity risk. **Conclusions**: These findings identify compound 1a as a promising lead, while compounds 1b and 1c remain viable candidates for further optimization. However, experimental validation is critical to confirm the predicted biological activity and safety profiles.

## 1. Introduction

### 1.1. Structural Characterization of Poly (ADP-Ribose) Polymerase (PARP)

**Figure 1:**
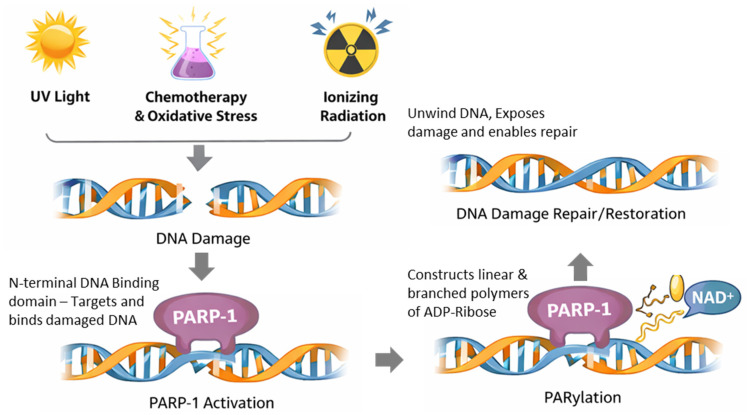
PARP1’s function in DNA damage and repair mechanism.

The Poly (ADP-ribose) polymerases (PARPs) superfamily consists of 18 members, which regulate various cellular functions such as cell proliferation, DNA recombination and repair, apoptosis in ischemic condition and necrotic death [1]. Among this superfamily, PARP-1 accounts for over 90% of ADP-ribosylation in cells and is thus the most abundant and well-studied enzyme [2,3]. Furthermore, sequence similarity tests across different species were performed against PARP members and indicated that PARP1 has highly conserved active site sequences for humans, rats and mice, which makes it ideal for investigations [4]. In other studies, PARP1 has been shown to be very evolutionarily conserved in eukaryotes and a well-known ADP-ribosyl transferase that cleaves nicotinamide adenine dinucleotide (NAD+) into ADP-ribose and nicotinamide. During this process PARP further transfers the ADP-ribose units onto a variety of proteins such as histones, DNA polymerases, topoisomerases and DNA ligases or themselves [5,6,7]. The whole PARP superfamily shares a conserved catalytic domain, known as the PARP signature [2]. However, PARP-1 is a 113 kDa protein which has a well-preserved structural and functional organization characterized by four major domains, namely the N-terminus DNA binding domain with two zinc fingers (42 kDa), the central auto modification domain (16 kDa) and the C-terminus catalytic domain (55 kDa) [8,9,10,11]. The N-terminus DNA binding domain functions by targeting and binding to damaged DNA single-strand breaks to trigger polymerization of ADP-ribose (Figure 1). This ultimately leads to the unwinding of the DNA from the positively charged histone proteins, thus exposing the damaged DNA to repair [11,12]. On the other hand, the catalytic domain functions by using NAD+ as a substrate to construct linear and branched polymers of ADP-ribose onto its targets, thus enabling it to perform a pivotal role in DNA damage repair [12].

### 1.2. Poly (ADP-Ribose) Polymerase (PARP) as a Drug Target

As part of the PARP superfamily, PARP-2 is a structural homolog of PARP-1 and exhibits similar functions. However, its DNA binding domain is different and does not contain the central auto-modification domain. Both PARP-1 and PARP-2 (to a lesser degree) function as DNA damage sensors by binding with high affinity to the site of single- and double-strand DNA breaks. Therefore, the overexpression of PARP-1 in various cancers, particularly that of the breast and ovaries, has been linked to the overall prognosis in cancers [3,8,9]. Considering the key role played by PARP-1 in terms of maintaining genomic integrity by repairing single-stranded DNA lesions, PARP-1 has emerged as an attractive therapeutic target in carcinogenesis anchoring DNA damage. These DNA lesions are mostly caused by UV light, ionizing radiation, chemotherapy or products of cellular and oxidative metabolism [3,13]. A graphical presentation of how external agents induce DNA damage, leading to the activation of PARP-1, PARylation and ultimately repair/restoration of the DNA, is shown in Figure 1.

Therefore, the PARP-1 catalytic site is largely targeted by inhibitors which seek to exploit the synthetic lethal dependency of *BRCA1/2* mutated cancer cells. This mechanism enables the selective killing of cancer cells whilst preserving normal ones.

### 1.3. Cytotoxicity of the Current PARP-1 Inhibitors

**Figure 2:**
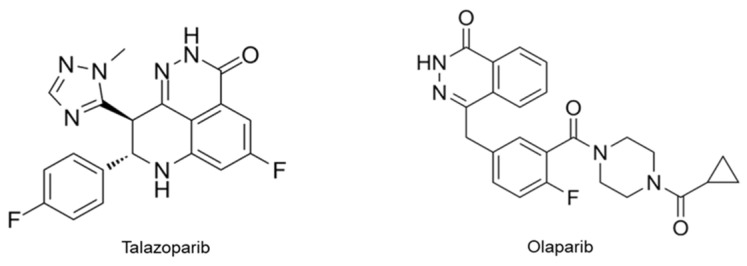
FDA-approved PARP inhibitors for the treatment of breast cancers.

Preclinical studies have revealed that cell lines with deficient *BRCA1* and *BRCA2* were 1000-fold more sensitive to PARP inhibition compared to wild-type or heterozygous mutant cells [14,15]. As *BRCA1* and *BRCA2* are well-known tumor suppressor genes whose mutations are associated with breast, ovary, prostate and pancreas cancers, this finding has therefore strengthened the significance of PARP-1 inhibition in cancer [14,15]. Multiple studies ranging from both in silico and in vitro approaches have made significant strides in attempts to design small molecular inhibitors of the PARP enzyme, resulting in the FDA approval of Talazoparib and Olaparib (Figure 2) [16,17].

However, there are side effects reported with several of the polymerase inhibitors, and the most common one is their off-target interactions with cardiac ion channels, particularly the human ether-à-go-go 1 (hERG1) channel [18]. This off-target ultimately led to severe cardiotoxicity that was also noted during the ABRAZO trial; even though Talazoparib exhibited strong inhibition of PARP compared to other drugs, its use was discontinued due to observed toxicity in some patients, where one suffered from anemia and at least two patients had liver test abnormalities [19]. Additionally, a high proportion of patients (almost 30%) needed blood transfusions due to symptomatic anemia during treatment. As the patients were divided into two cohorts (49 and 35 patents respectively), Grade 3 anemia was observed in 33% of the patients in cohort 1 and in 40% of patients in cohort 2 [16,19]. These side effects are currently managed by reducing the administered dosages, which work for some patients, but others still experience significant cytotoxicity problems. It is also worth noting that too much reduction in the drug concentration, particularly below its IC_50_, can also negatively impact the efficacy of the drug.

### 1.4. PARP-1 Mechanism of Action and Inhibition

PARP-1 senses DNA damage and binds to DNA breaks, synthesizing Poly (ADP-ribose) (PAR) chains on target proteins (i.e., PARylation) adjacent to the DNA break and itself (i.e., autoPARylation). These PAR chains lead to the recruitment of additional DNA repair effectors that complete the DNA repair process, thus enabling PARP-1 to act as a signal transducer. In its non-DNA bound state, PARP-1 has limited catalytic activity because of the auto-inhibitory effect caused by the interaction of the helical domain (HD) with its catalytic domain [20]. When PARP-1 targets and binds to the DNA via zinc finger domains, a conformational change in the PARP-1 protein relieves the auto-inhibitory interaction between the HD and the catalytic domain, allowing nicotinamide adenine dinucleotide (β-NAD+), the PARP-1 co-factor, to bind to the active site of the enzyme. This binding of β-NAD+ enables it to be hydrolyzed by PARP-1, thus catalyzing the transfer of ADP-ribose moieties known as PARylation. This process ultimately mediates DNA repair through chromatin modification (e.g., via histone-PARylation) and by recruiting DNA repair effectors. PARP-1 eventually undergoes autoPARylation which enables it to detach and leave the DNA damaged site [21].

**Figure 3:**
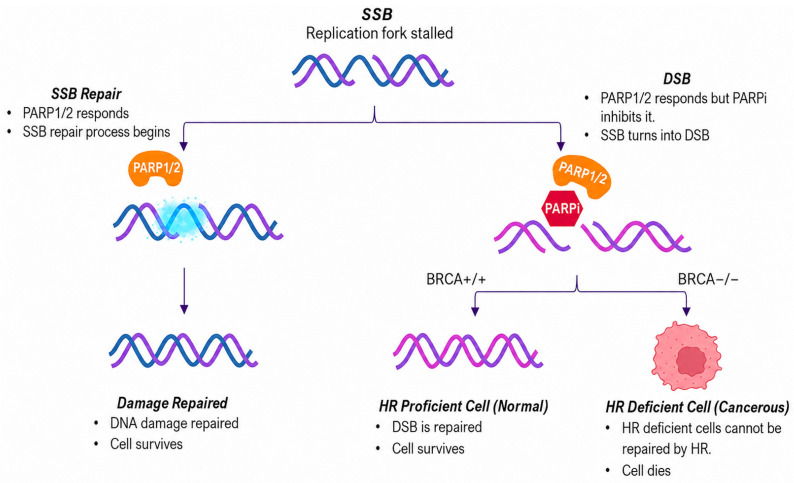
The synthetic lethality mechanism in *BRCA1/2*-deficient cells and PARP inhibition. The left-side shows PARP1/2 (Orange) binding onto the single strand break (SSB) and recruiting other enzymes (blue cloud) to facilitate DNA repair. However, the right-side illusrates an introduction of a PARP inhibitor (red) which stalls the replication fork creating a double strand break (DSB). Normal cells still facilitate repair via homologous recognition (HR) due to non-mutated genes (BRCA+/+), whilst cancer cells die due to mutation (BRCA−/−) in HR genes. Adapted from [23].

In the absence of PARP-1 inhibition, cancer cells can repair damaged DNA breaks through PARylation and autoPARylation and continue to proliferate. However, upon PARP inhibition the binding of β-NAD+ on the active site is inhibited, which prevents PARylation and autoPARylation [22]. This ultimately facilitates the trapping of PARP-1 on damaged DNA [22]. This trapping stalls or delays the progress of the replication fork, turning single-strand breaks (SSB) into double-strand breaks (DSB) (Figure 3) [23]. Most importantly, it is worth noting that normal cells can restore these delayed replication forks through homologous recognition repair (HRR). However, cancer cells lack one of the key HRR proteins, such as *BRCA1*, *BRCA2*, *PALB2* or *RAD51*. Alternative DNA repair mechanisms are used in attempts to repair DNA lesions caused by PARP inhibition; this is done through non-homologous or microhomology-mediated end-joining [21]. However, these mechanisms are prone to errors and can lead to the fragmentation of the DNA which ultimately kills the cell (Figure 3).

Therefore, this study seeks to exploit the synthetic lethality framework to design a new set of PARP-1 inhibitors for breast cancer treatment. This was achieved through a comprehensive implementation of computational chemistry techniques to aid the design and preclinical processing of safer and highly effective drug candidates. The process involved screening ultra-large chemical libraries using AIDDISON^TM^ v2023 followed by a virtual screening workflow comprising three docking regimes, namely High-Throughput Virtual Screening (HTVS), Standard Precision (SP) and Extra Precision (XP). Additionally, the in vitro activity data (Olaparib (IC_50_ = 5 nM), Rucaparib (IC_50_ = 7 nM), and Talazoparib (IC_50_ = 1 nM)) of the current therapies were also considered during the design process [23].

## 2. Results and Discussion

### 2.1. Molecular Docking Protocol Validation

**Figure 4:**
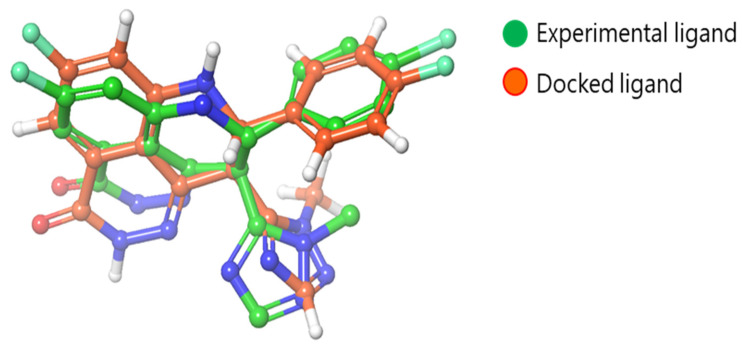
Validation of the docking protocol.

To confirm the accuracy of the molecular docking procedure, the redocking experiments were performed by removing the co-crystallized Talazoparib ligand from the binding site of the PARP-1 crystal structure and redocking it back to the same active site of the protein. The superposition in the figure shows the position of the crystallographically identified Talazoparib ligand (green) and the position of the ligand after redocking (orange) (Figure 4). The high degree of similarity between the two positions clearly shows the effectiveness of the docking program. The RMSD between the position of the docked ligand and the experimentally (crystallographic) elucidated one is 0.995 Å (Figure 4). An RMSD of less than 2 Å for a docked ligand compared to its experimental position is considered to be an excellent agreement and therefore good for confirming the accuracy of the docking protocol [24], implying that the method used can effectively reproduce the experimentally determined ligand’s binding modes.

Therefore, this calculated RMSD value (0.995 Å) indicates the effectiveness of the docking parameters and scoring functions used. In addition, the almost perfect overlap of the redocked and crystallographic conformations within the catalytic domain of PARP-1 suggests optimal grid preparation, ligand protonation and/or charging as well as the docking settings. Consequently, this validated docking workflow provides confidence that the subsequent docking results obtained for the designed compounds (1a–1c) are credible and structurally meaningful, thereby supporting their comparative evaluation against the reference inhibitor Talazoparib and reinforcing the reliability of the computational screening process.

### 2.2. Structure-Based Virtual Screening and Molecular Docking Analysis

Molecular docking studies indicated enhanced binding affinity of the designed compounds (1a–1c) as compared to the reference inhibitor, Talazoparib. This Talazoparib inhibitor showed a docking score (XP) of −6.778 kcal/mol with an identical XP GScore of −6.778 kcal/mol and a Glide emodel of −57.728 kcal/mol. However, for compound 1a the docking score was observed to be −9.488 kcal/mol and the XP GScore was −9.715 kcal/mol along with a Glide emodel of −70.279 kcal/mol. The docking scores for compounds 1b and 1c were found to be −9.349 kcal/mol and −9.255 kcal/mol with corresponding XP GScores of −9.500 kcal/mol and −9.437 kcal/mol respectively. Furthermore, compound 1b showed the best Glide emodel value of −71.371 kcal/mol when compared to the reference inhibitor Talazoparib, which has a Glide emodel value of −62.141 kcal/mol (Table 1).

Altogether, the three designed compounds gave more negative docking scores and XP GScores than Talazoparib, which indicates greater affinity towards the target protein. Compounds 1a and 1b have higher docking scores by a magnitude of approximately 3 kcal/mol when compared to Talazoparib with further greater Glide emodel energy values. Compound 1c has the least favorable emodel value amongst the top hits. However, it is far better than the reference compound in all scoring parameters. Therefore, compounds 1a–1c have indeed been shown to have better predicted binding energy and stability towards the target protein than Talazoparib and His862, Gly863 and Ser904 are the three critical residues, forming hydrogen bonds as well as π-π stacking interactions that facilitate the binding coordination (Table 1). Hence, the current docking studies indicate that 1a–1c are good lead compounds that deserve further investigation.

**Figure 5:**
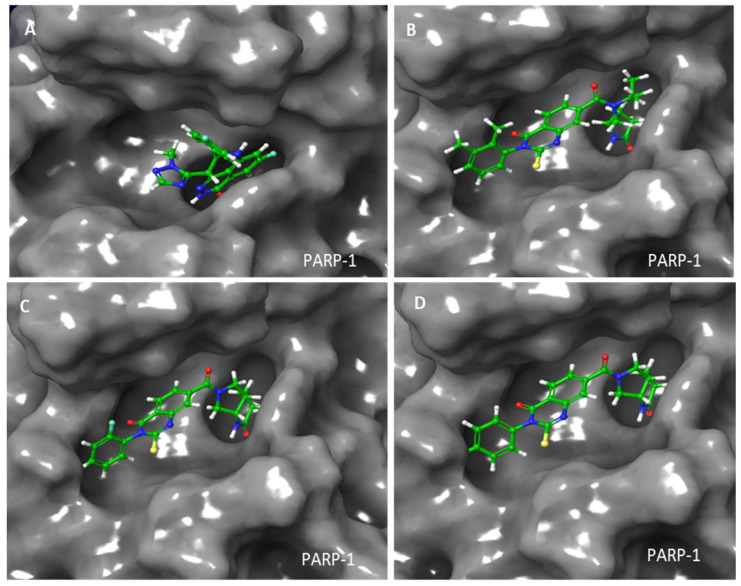
(**A**–**D**) A gray surface representation of the Poly [ADP-Ribose] polymerase 1 catalytic domain complexed with Talazoparib and compounds 1a, 1b and 1c.

A gray surface representation of PARP-1 complexed with Talazoparib and compounds 1a, 1b and 1c is shown in Figure 5. During the virtual screening process compounds 1a–1c emerged as top hits with better binding energies than the FDA-approved Talazoparib inhibitor. This study implicates these compounds as good potential inhibitors for the PARP-1 protein, particularly for the treatment of breast cancer. Talazoparib is seen to adopt a more localized binding mode within the active site, whilst the top three hits assume similar yet occupy a wider surface area forming multiple hydrophobic and polar interactions within the active site to facilitate the binding coordination (Figure 5). However, these docking findings are still preliminary and need further experimental validation.

### 2.3. Molecular Dynamics Analysis

To determine the thermodynamic stability of the complexes, four molecular dynamic simulation studies were conducted with a particular focus on the root mean square deviation (RMSD), root mean square fluctuation (RMSF), protein–ligand interactions in terms of ionic bonds, hydrophobic interactions, hydrogen bonds and water bridges as well as protein–ligand contacts (PL-Contacts) throughout the simulation. The Desmond system builder tool in Maestro v2023-2 was used to construct the molecular dynamic solvation systems of all four drug complexes. Subsequently, the molecular dynamic studies were executed for 100 ns and subsequently extended to 250 ns, focusing on the top designed hit whilst monitoring the interactions.

#### 2.3.1. Analysis of the Root Mean Square Deviation (RMSD) Profiles

**Figure 6:**
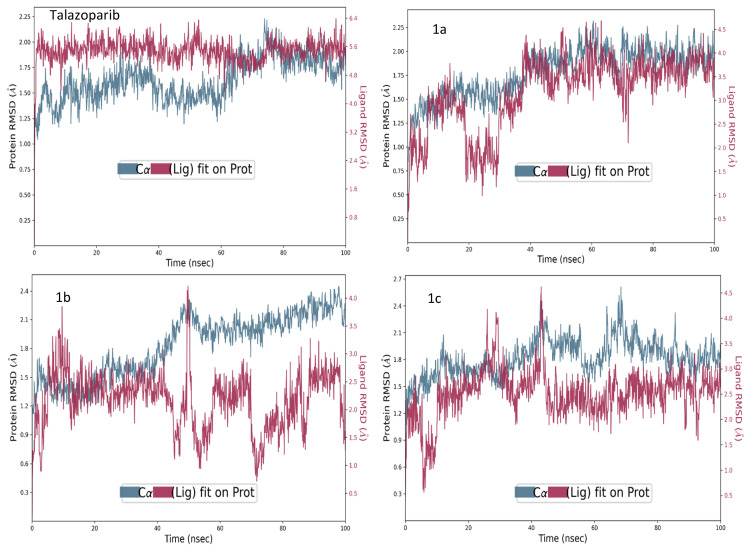
Molecular dynamic studies showing RMSD data of the Poly [ADP-ribose] polymerase 1 catalytic domain complexed with Talazoparib and compounds 1a, 1b and 1c.

Notably, the RMSD data for compound 1a depicts an excellent and consistent alignment with the Cα atoms of the Poly [ADP-ribose] polymerase 1 catalytic domain (Figure 6). It is also worth mentioning that the binding of compound 1a–1c exhibits a more thermodynamically favorable interaction with lower RMSD ranges (2.5–3.6 Å) compared to the FDA-approved Talazoparib (5.6 Å) (Figure 6). Furthermore, compound 1a has been proven to tightly bind PARP-1 better than Talazoparib with no degree or change in displacement, as observed by its proximity to PARP-1’s Cα atoms for a greater extended period in the simulation. Hence, this implicates a very good drug residence time which is a critical feature in determining the specificity and effectiveness of drug candidates.

#### 2.3.2. Analysis of the Root Mean Square Fluctuation (RMSF) Profiles

**Figure 7:**
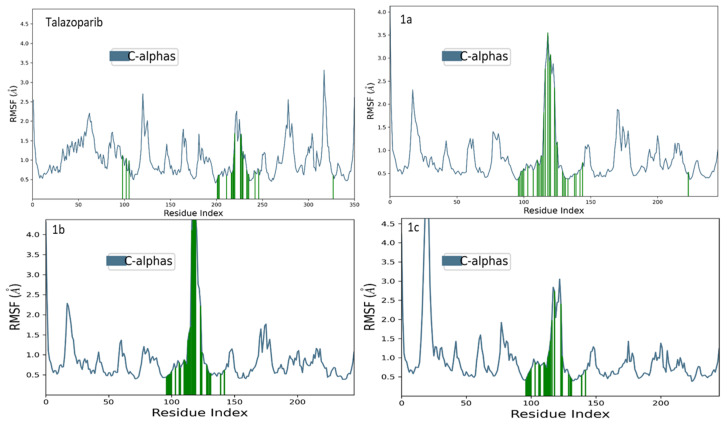
This figure represents the characterization of the local fluctuation of the protein’s alpha carbons (P-RMSF) with the protein–ligand contacts shown in green vertical lines for Talazoparib and compounds 1a, 1b and 1c respectively.

Root mean square fluctuation (RMSF) analysis was performed to evaluate the flexibility of each protein residue in the complexes with Talazoparib and the designed compounds 1a–1c (Figure 7). The RMSF profiles of all four systems show that almost all the residues have fluctuation values within a comparable range (less than ~1.5 Å), suggesting that the protein is maintained in a similar conformation and the global stability is not significantly affected by the different ligands. In the Talazoparib-bound complex, some regions show moderate fluctuations, especially in the loop regions located far from the active site and at residue indices ~200–240 and ~300–320. The residues involved in ligand–protein interactions are marked with green bars and are located in stable regions of the protein (low-to-moderate fluctuation values) (Figure 7).

**Figure 8:**
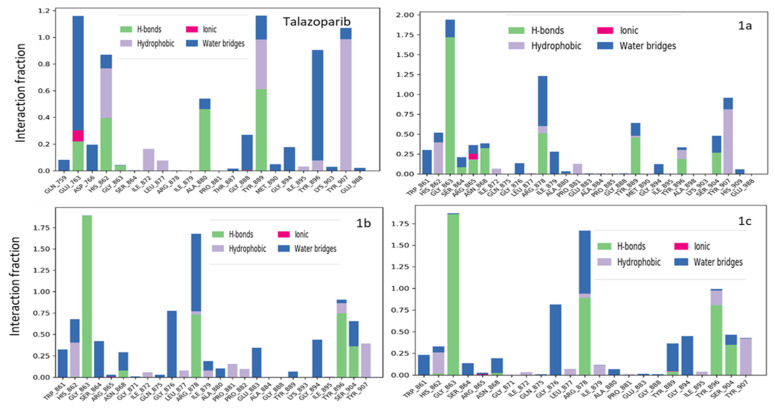
Interaction fraction of the docked compounds with the Poly [ADP-Ribose] polymerase 1, in which the hydrogen bonds, hydrophobic and water bridge interaction dominate the binding co-ordination.

For the complexes with compounds 1a–1c, the overall RMSF patterns are broadly comparable to that observed for Talazoparib, with most residues exhibiting fluctuations below ~1.5 Å. However, some localized differences are observed near the binding site region (~95–130). Compound 1a shows a moderate peak around ~115–120, while compound 1b displays a more pronounced but highly localized fluctuation in a similar region. In the 1c complex, a noticeable peak is observed near ~125–130, but this does not affect the surrounding residues, thus they remain relatively stable. Therefore, we note that the observed fluctuations do not represent a general destabilization of the entire protein structure. This is shown in Figure 7 where the profiles of local movements for each protein patch of the protein–ligand complex have been examined. The vast majority of the residues identified to be involved in the interaction with the ligands correspond to regions having moderate RMSF values. This indicates that the protein–ligand interactions are well retained throughout the entire duration of the molecular dynamic simulations. As shown in Figure 8, the RMSF values corresponding to compounds 1a, 1b and 1c are quite similar to each other, and not significantly different from those obtained for Talazoparib. This suggests that, at least within the duration of the simulations, the binding of these compounds to the target protein does not introduce remarkable structural fluctuations and thus they deserve further experimental evaluation.

#### 2.3.3. Interaction Fraction Analysis

Interaction fraction analysis revealed the occurrence and stability of ligand–protein interactions and that our designed compounds 1a–1c exhibit a greater affinity and stability for the binding site of PARP-1 compared to Talazoparib. Similarly to previous studies [25,26], Talazoparib adopted the same binding mode within the PARP-1’s catalytic domain and was involved in hydrogen bonding, hydrophobic and water bridge interactions with Glu763, His862, Ala880, Tyr889, Tyr896 and Tyr907 (Figure 8). While these interactions did occur, a large portion of interactions between Talazoparib and the catalytic domain were contributed by water-mediated contacts suggesting limited occurrence of direct protein–ligand interactions. In contrast, the interaction fractions of 1a–1c were greater and encompassed more catalytic domain residues. A more close-up view of compounds 1a–1c in the binding site is shown in Figure 8, with high fractional occurrence of hydrogen bonding (H-bonds) to Gly863. In addition, Arg878 is also prevalent to only compounds 1a–1c and constitute a combination of H-bonding and minimal water bridge interactions. All designed compounds have other stabilizing contacts present, which include His862, Ser904, Tyr889 and Tyr907 consisting of hydrophobic interactions and further hydrogen bonding interactions for compound 1a. Similarly, compound 1b also has hydrophobic, H-bonds and some water-mediated contacts with Gly876, Tyr896, Ser904 and Tyr907. The interaction pattern for 1c is very similar to that of 1b with additional hydrophobic, H-bonds and water-mediated contacts with Gly876, Tyr896, Ser904 and Tyr907. In total, the greater interaction fractions for 1a, 1b and 1c as well as the wide variety of contact types suggest greater stability of the ligand–protein complexes and are hence in line with their expected properties as good potential PARP1 inhibitors (Figure 8).

**Figure 9:**
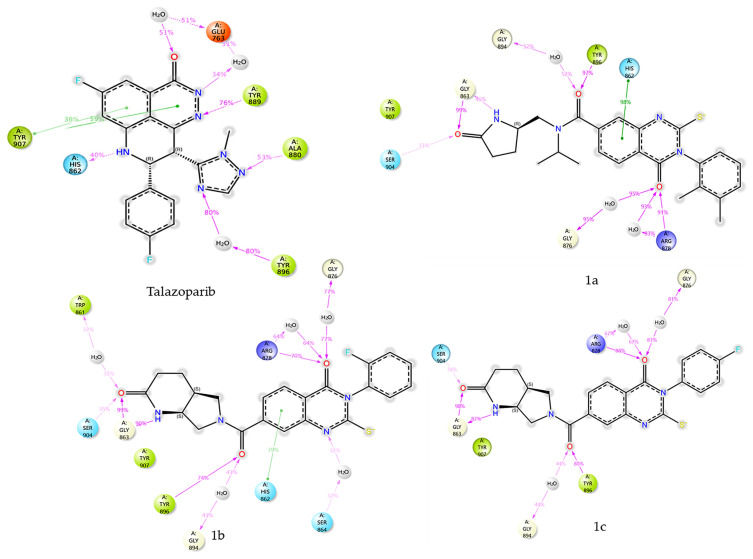
A detailed ligand interaction with the protein residues at 30% simulation time.

The 30% MD trajectory ligand interaction diagrams of the PARP-1’s active site residues with Talazoparib and compounds 1a–1c are shown in Figure 9. These diagrams provide a snapshot of the intermolecular contacts that stably exist between the active site residues, Talazoparib and the designed compounds at various regions of the binding pocket. Here, the hydrogen bonds, water molecules and hydrophobic interactions that exist for at least 30% of the simulation time are shown, and the total percentage of the existence of such interactions for the corresponding species and atom pairs are depicted. Based on PARP-1’s active site, the 30% ligand interaction diagram of Talazoparib has hydrogen bonds to Tyr889, Tyr907 and Ala880 residues as well as water molecules to Glu763 and Tyr896. All these contacts are consistent with reported structures of PARP-1 inhibitors binding to the active sites [25,26]. However, all the contacts are somewhat localized to specific parts of the ligands. Interestingly, compound 1a not only retains hydrogen bond contacts to Gly863 of the enzyme as observed with 1b and 1c, but it also retains one water bridge interaction to Arg878, facilitating its correct orientation at the catalytic site. Meanwhile, the aromatic ring part of compound 1a was also in close proximity to Tyr907 and Trp861 residues, the latter two having been proven to be crucial to the activity of PARP-1.

Interestingly, compound 1b also established multiple stable contacts with several catalytic site glycine and aromatic residues (Gly863, Arg878, Tyr896, Tyr907 and His862). This was achieved both through direct hydrogen bonds and the presence of water molecules. Similarly to 1b, compound 1c also established an interaction profile that comprised hydrogen bonds and water-mediated contacts to catalytic site residues, including Gly863, Tyr896 and Arg878. The 30% interaction analysis revealed that all the designed compounds establish a larger number of stable contacts to the catalytic site residues of PARP-1 when compared to Talazoparib. These interactions are generally broader and more distributed over the catalytic site, with compounds 1a, 1b and 1c establishing in some cases multiple hydrogen bonds to the catalytic site residues, as well as multiple water-mediated contacts, and stable contacts to several glycine and aromatic residues which is consistent with other studies [23]. These observations suggest that all the designed compounds are stable in the catalytic site and thus deserve further in vitro and in vivo experimental validation.

#### 2.3.4. Protein–Ligand Contact Analysis

**Figure 10:**
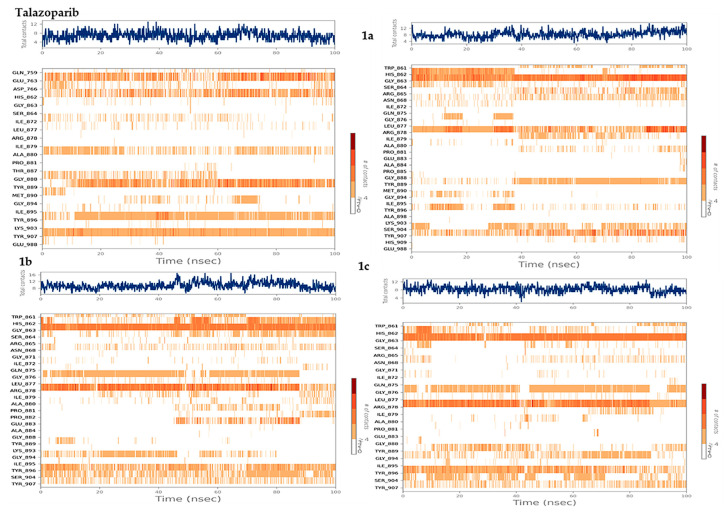
Protein–ligand contact analysis with the protein residues over 100 ns simulation time. The density of these interactions is represented by the side-bar on the right-hand side of the each graph.

The protein–ligand contact analysis in Figure 10 shows that the four compounds maintain ongoing interactions within the PARP-1 binding site. However, they each have distinct interaction patterns with individual residues rather than adopting the same binding mode. While the majority of Talazoparib stabilization comes from a set of residues such as Glu763, His862, Tyr896 and Tyr907, some contacts to Ala880 and Tyr889 are also observed. However, compounds 1a–1c exhibit persistent contacts with Gly863 and Arg878 which encore their stability within the active site. Additionally, they consistently interact with residues His862, Ser904 and Tyr907 (Figure 10). Furthermore, compounds 1b and 1c also interact with Gly876 and Tyr896 residues forming interactions that are long-lived throughout the simulation. While these ligands do not display the evenly balanced contacts observed for Talazoparib, they achieve a similar level of stabilization by compensating for weaker contacts to one set of residues with stronger and more continuous contacts to Gly863 and Arg878, mediated by other side chain interactions (Figure 10). This indicates that there are multiple stabilization pathways within the active site rather than a single optimal interaction pattern.

#### 2.3.5. Ligand Property Profile Analysis

**Figure 11:**
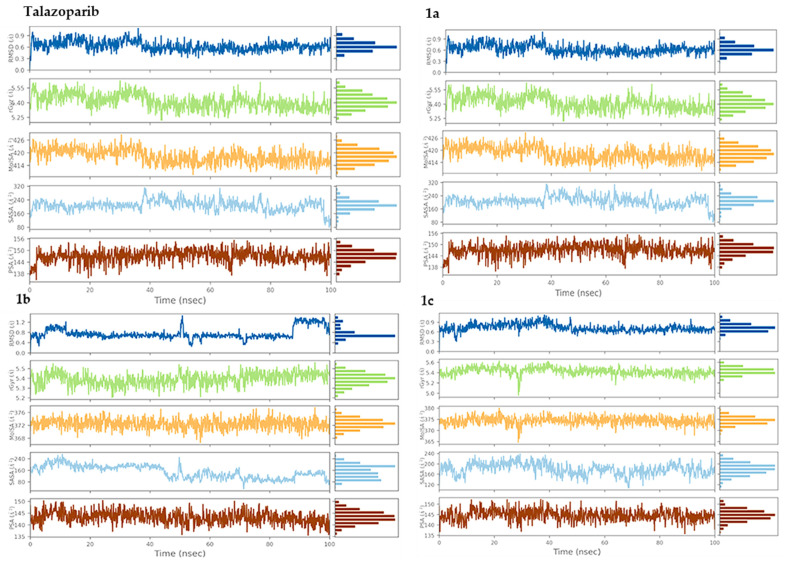
Ligand property profile analysis of Talazoparib and the top 3 potential inhibitors over a 100 ns simulation period.

The ligand property profiles show that all the studied compounds remain stable with subtle differences in compactness and adaptability. RMSD traces (top) indicate that all ligands reached rapid equilibrium within 10–20 ns and remained stable within a ~2 Å fluctuation range except for 1b, which showed a late, minor fluctuation indicating occasional conformational changes within the binding site. The radius of gyration (rGyr, second row) remained stable along the trajectories, showing that the ligands maintained consistent compactness. Some variation in molecular surface area (MolSA, third row) and solvent-accessible surface area (SASA, fourth row) was observed, where 1b showed the most buried conformational state (decrease in SASA). However, the fluctuations in MolSA and SASA were mostly subtle and indicate small differences in how the ligands adapt their surfaces during binding. The percentage of polar surface area (PSA, bottom) remained relatively stable and indicates that the studied compounds maintained consistent and favorable polar interactions with the receptor throughout the simulations. Overall, the ligands remained stable and showed broadly similar properties, yet their specific conformations within the binding pocket show different dynamic behaviors (Figure 11).

### 2.4. Molecular Mechanics–Generalized Born Surface Area Analysis

The binding free energy (ΔGbind) values provide an overall estimate of ligand affinity towards the target protein, where more negative values indicate stronger binding. The FDA-approved reference inhibitor Talazoparib exhibited a ΔGbind of −63.734 kcal/mol. Among the investigated hits, compound 1a demonstrated the most favorable binding affinity with a ΔGbind of −67.820 kcal/mol, which is more negative than that of Talazoparib, suggesting stronger predicted binding to the target site. However, compounds 1b (−61.573 kcal/mol) and 1c (−61.329 kcal/mol) displayed lower yet comparable binding energies with Talazoparib, indicating slightly similar binding strength with the reference, but also possible room for lead optimization (Table 2).

The energy components show which individual parts are responsible for the different affinities. Compound 1a shows significantly higher van der Waals (ΔG_VdW = −66.146 kcal/mol) and lipophilic (ΔG_Lipo = −27.652 kcal/mol) interaction energies in comparison with that of Talazoparib (−50.041 kcal/mol and −20.548 kcal/mol respectively). Hydrophobic as well as dispersion interaction energies are found to be the most dominant and may be the reason for better stabilization of the ligand in the protein binding pocket. In addition, compound 1a also shows higher Coulombic (electrostatic) as well as higher hydrogen bond interaction energies in comparison with that of Talazoparib. However, the packing energy (−4.211 kcal/mol) of compound 1a is the only lower-energy component in comparison with that of Talazoparib (−7.591 kcal/mol). But in total the energy gained from the van der Waals and lipophilic interaction energies is more dominant than the packing energy. Therefore, compound 1a shows a higher predicted binding affinity in comparison with Talazoparib (Table 2). The energy components of compound 1b and 1c show slightly lower overall binding energies in comparison with Talazoparib. These energy components may vary, but the large unfavorable contribution of solvation energy and lesser hydrophobic interaction is the cause of the reduced ΔG_bind, but can be easily improved through lead optimization. Of all the compounds, 1a shows maximum binding affinity and therefore a better hit amongst the evaluated compounds. However, all the three top compounds, namely 1a–1c, remain potential drug inhibitors that should be optimized to enhance their binding energy, and they warrant further in vitro and in vivo investigations.

### 2.5. Extended Molecular Dynamics Stability Analysis of the Top Hit and Talazoparib

**Figure 12:**
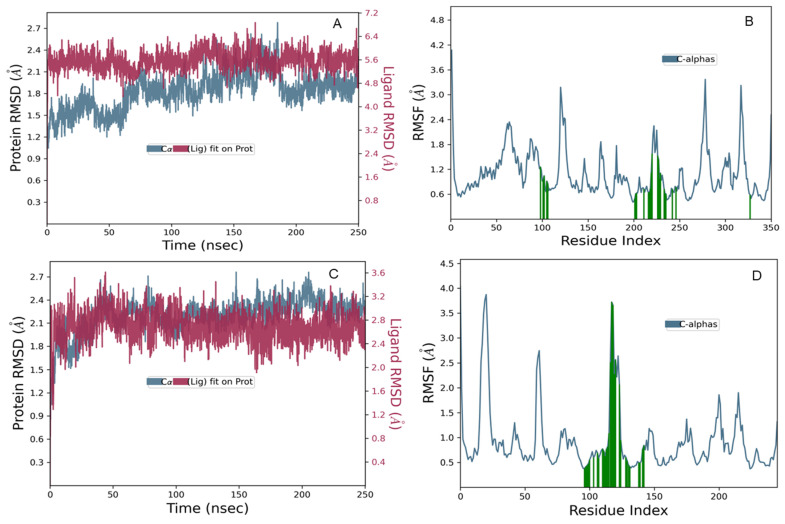
Illustration of an extended molecular dynamics simulation study for compound 1a and Talazoparib. (**A**,**B**) show Talazoparib’s RMSD and RMSF data whilst (**C**,**D**) indicates the same RMSD and RMSF data for compound 1a respectively. The interacting residues within the active site are represented in green vertical-lines on the both RMSF graphs.

The 250 ns extended MD simulations were performed only for the complexes of compound 1a and Talazoparib with PARP-1. Compound 1a was selected as the most promising candidate among the designed compounds, presenting the highest docking score and the most favorable MM/GBSA-based free energy of binding. As shown in Figure 12A,C, the two simulated complexes remained stable along the entire simulation time. The protein backbone RMSD values remained below 2.5 Å for both systems, indicating that both complexes were well accommodated in the PARP-1 active site. The ligand RMSD values instead highlighted some significant differences between the two ligands. In particular compound 1a presented values of ligand RMSD fluctuating between 2.4 Å and 3.2 Å, whereas Talazoparib presented values fluctuating between 5 Å and 6 Å. This data suggests that compound 1a maintained a fixed binding mode within the active site of PARP-1 along the entire simulation time, whereas Talazoparib presented some degree of flexibility. However, both systems did not present any major conformational change or even dissociation of the ligand along the entire 250 ns simulation period.

The RMSF analysis was also performed for both complexes as shown in Figure 12B (Talazoparib) and Figure 12D (compound 1a) to examine the stability of both structures. While fluctuation at the loop and terminal ends are expected to be large, fluctuations in the rest of the protein outside the core catalytic site were indeed localized. In contrast, Talazoparib exhibited large fluctuations across several residue clusters, while 1a displayed relatively localized fluctuations centered at residue clusters ~100–140 with the rest of the protein backbone stable. These findings strongly support our 100 ns results of long-timescale simulations where 1a exhibited the most stable binding within the active site of PARP-1 amongst the designed hits. Altogether, compound 1a has been shown to bind better and more thermodynamically favorably than Talazoparib, as predicted throughout the 250 ns simulation, and therefore warrants further experimental validations.

### 2.6. Stability Assessment via Quantum Optimization and DFT Calculations

**Figure 13:**
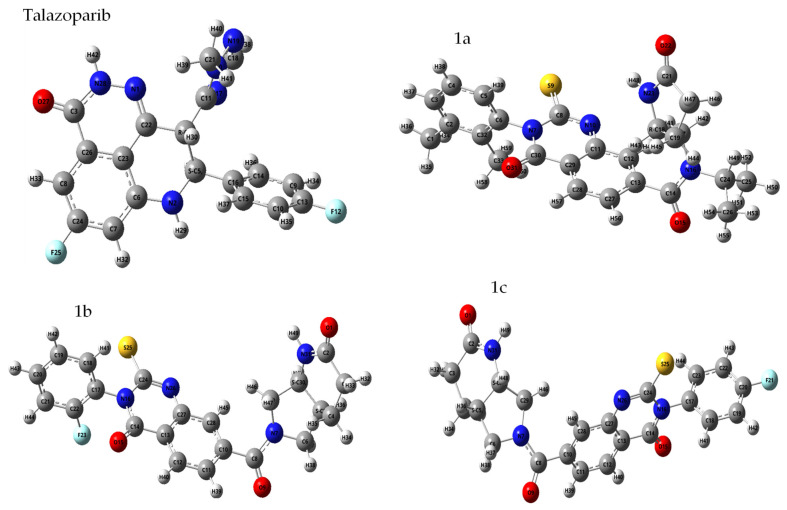
Geometry optimization of Talazoparib and the three top candidates.

Based on the quantum optimization study depicted in Figure 13 it can be inferred that 1a–1c have an optimized structure favorable for their interactions with PARP-1 in comparison to Talazoparib. Talazoparib has a relatively small core with low heteroatom density versus 1a, 1b and 1c, all of which possess additional functional groups integrated into the same binding site, including heteroatoms (nitrogen, oxygen and sulfur) strategically located for optimal affinity as well as larger aromatic rings that participate in increased π-π and hydrophobic interactions within the binding site. The presence of sulfur in 1a–1c and the extra polar atoms compared to Talazoparib suggest a higher electrostatic interaction potential and stronger van der Waals binding to the core of PARP-1’s binding site atoms. Therefore, supporting the notion in Figure 13, compounds 1a–1c will be worthy of a more detailed experimental investigation as new PARP-1 inhibitors.

**Figure 14:**
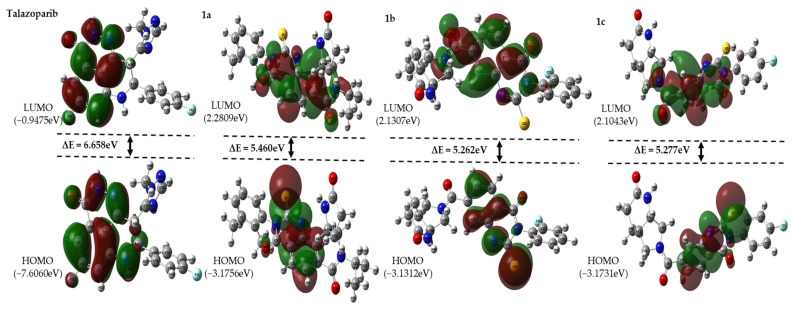
Geometry optimization and Frontier Molecular Orbital (FMO) Analysis. The green and red zones represents positive and negative wave function phases, which corresponds to the electron donor and acceptor regions respectivity.

The Frontier Molecular Orbital (FMO) analysis presented in Figure 14 provides information on the electronic properties and reactivity of Talazoparib and related small molecules (1a, 1b and 1c) in the present study. The figure indicates that the HOMO of Talazoparib is mainly distributed over the core of the heteroaromatic rings and the heteroatoms at the edges, and the LUMO is located over the more electron-deficient regions of the molecule. These charge distributions facilitate the transfer of charges within the molecule and confirm Talazoparib’s inhibitory activity.

For the designed compounds, the HOMO and LUMO contain almost the same degree of delocalization as the pharmacophore components, which is favorable for protein–ligand (P–L) interaction. In compound 1a, the HOMO is highly delocalized over the whole aromatic moiety and heteroatom-containing groups, while the LUMO is delocalized over the central scaffold and substituents. The charge transfer ability of compound 1a, which is due to the strong electron-donating and electron-withdrawing ability, is supported by a suitable HOMO–LUMO gap, which indicates a balanced electronic configuration that maintains molecular stability while supporting adequate chemical reactivity. This signifies an improvement relative to Talazoparib and is not limited to compound 1a, but can also be observed on 1b and 1c (Figure 14). The HOMO and LUMO of compound 1b are localized on different parts of the structure. The HOMO is delocalized over the aromatic part, while the LUMO has some extension toward the electron-withdrawing groups. This clearly shows the donor–acceptor characteristics of the system, which is favorable for charge-transfer interaction with amino acids within the catalytic pocket. The localization of the orbitals is a little higher than that of Talazoparib, but it should not prevent compound 1b from exhibiting sufficient flexibility for effective P–L interaction.

The FMOs and energy levels of compound 1c are also displayed in Figure 14. It can be seen from this figure that the HOMO of 1c is localized in the heteroatom-containing rings, and the LUMO covers both the conjugated rings and the substituents in adjacent sites. This electronic arrangement suggests that the charge transfer between the two sites happens through an intramolecular process, and it implies a high probability of intermolecular electronic interactions with the protein. Moreover, the energy gap between the HOMO and LUMO of 1c has been shown to be within the same range as that of 1a, 1b and Talazoparib, which indicates that compound 1c possesses both the necessary stability and reactivity required in a chemical inhibitor. As illustrated in Figure 14, the FMO results show that the designed compounds 1a–1c possess electronic features similar to or even better than that of the known inhibitor Talazoparib due to the displayed balance between stability and reactivity. From the orbital distribution and energy gaps of the HOMO and LUMO, it is found that compounds 1a–1c have favorable electronic features for efficient charge transfer and optimal protein–ligand binding in the active site of PARP-1. According to these findings and the previously discussed binding affinities to PARP-1, all designed compounds (1a–1c) have great potential for further investigations (Figure 14).

The DFT descriptor comparison in Table 3 indicates that compounds 1a–1c have better electronic properties than that of Talazoparib, which should lead to higher reactivity of the molecules and better affinity with the target protein. Starting from the HOMO–LUMO energy gaps (ΔG), all three lead molecules showed smaller values (ΔG ≈ 5.26–5.46 eV) when compared with Talazoparib (6.659 eV), confirming their better electronic mobility along with their ability to transfer charges more efficiently during protein–ligand binding events. Similarly, smaller hardness values (η) and larger softness values (δ) were obtained for 1a, 1b and 1c (η ≈ 2.63–2.73 eV; δ ≈ 0.367–0.381 eV) when compared with those of Talazoparib (η = 3.330 eV; δ = 0.300 eV), confirming the better adaptability of our three lead molecules to the specific electronic environment of the target protein (PARP-1) binding site (Table 3). Moreover, higher values of global electronegativities and slightly higher values of global electrophilicities were obtained for compounds 1a–1c confirming their better electron affinity properties, along with better mutual stabilization of their possible intermolecular interactions. Therefore, these findings align with the docking and molecular dynamics data discussed in Section 2.2 and Section 2.3, where these lead compounds (1a, 1b and 1c) displayed better reactivity and protein–ligand binding affinities compared to Talazoparib.

### 2.7. Pharmacokinetic (ADMET) Analysis

Based on the ADMET data illustrated in Table 4, it can be inferred that the three designed compounds (1a–1c) possess better pharmacokinetics and safer profiles than Talazoparib, which means they possess better drug-likeness and activity. All the designed compounds are within the acceptable ranges for the molecular weight, the number of hydrogen bond donors and acceptors, the lipophilicity (QPlogPo/w) and aqueous solubility, which are major criteria for evaluating drug-likeness. In addition, as shown in Table 4, the predicted QPlogHERG values for the designed compounds 1a, 1b and 1c are −4.543, −4.625 and −4.605, respectively, which are less than that of Talazoparib (−5.143). More negative values represent more potential risks of hERG channel blockade; less negative values indicate smaller risks of cardiotoxicity [27,28]. Therefore, it can be inferred that compounds 1a–1c possess no cardiotoxicity risks, whilst Talazoparib is more consistent with toxicity problems as indicated in previous clinical studies [19]. In addition, the dipole moment of Talazoparib (0.00) is beyond the recommended range (1–12.5), which suggests that Talazoparib is poorly polarized and hence has few interactions in the binding site, which is further confirmed by the relatively small interaction fraction calculated during the molecular dynamics simulations in Section 2.3 and the much wider energy gap seen in Section 2.5. In summary, based on their balanced ADMET profiles and the predicted lower QPlogHERG toxicity values, it can be inferred that the designed compounds 1a–1c possess better pharmacokinetics and safer profiles than Talazoparib, indicating that they have potential for acting as active PARP-1 inhibitors (Table 4). Therefore, based on the obtained results further experimental validation studies are critical to confirm the pharmacokinetic safety and therapeutic effectiveness of these designed compounds.

#### 2.7.1. Comparative Toxicological Assessment Employing the ProTox-3.0 Server

Toxicity predictions for the designed compounds 1a–1c and Talazoparib were performed by using ProTox-3.0 and are summarized in Table 5. Compound 1a shows the best safety profile with the highest predicted LD50 value (1200 mg/kg) and with the lowest toxicity class (Class 3), indicating comparatively lower acute oral toxicity than the other compounds and Talazoparib. All designed compounds and Talazoparib are predicted to be inactive for cardiotoxicity, nephrotoxicity, carcinogenicity, immunotoxicity, mutagenicity and cytotoxicity. For hepatotoxicity, all compounds 1a–1c are predicted to be inactive whereas Talazoparib is to be active (0.63). Clinical toxicity was predicted to be inactive for compound 1a whereas compounds 1b, 1c and Talazoparib have shown an active predicted clinical toxicity potential. However, all compounds are predicted to be active for neurotoxicity with Talazoparib showing the highest neurotoxicity probability (0.92). In summary, the designed compounds, particularly compound 1a, show improved safety profiles as compared to Talazoparib and thus serve as potential candidates for further development as safer PARP-1 inhibitors. However, as with all computational predictions, these findings need to be validated by in vitro cytotoxicity assays, hepatotoxicity screening and in vivo toxicological studies.

#### 2.7.2. Comparative Evaluation of hERG-Mediated Cardiotoxicity Using Pred-hERG v5.0

As compound 1a has shown better predicted properties than all the designed compounds and Talazoparib, a further hERG liability assessment was then elucidated using Pred-hERG 5.0—https://predherg.labmol.com.br/ (accessed on 14 May 2026), comparing 1a and Talazoparib. The predicted results indicated that both compounds were classified by Pred-hERG 5.0 as consensus non-blockers, indicating that they exhibit little risk of causing cardiotoxicity through hERG block. Talazoparib received a higher binary non-blocker confidence score of 82.7% compared to compound 1a which received a score of 69.4%. However, the multiclass model provided additional information that Talazoparib is a moderate hERG blocker. The predicted pIC50 values for Talazoparib and compound 1a were found to be 5.374 and 5.267 respectively, which indicated that compound 1a has a lower predicted affinity for hERG when compared to Talazoparib. The fragment contribution maps for both compounds were also created using Pred-hERG 5.0. These maps were used to identify aromatic regions associated with non-blocker activity as well as regions that make contributions to predicted blocker activity. As summarized in Table 6, the fragment contribution mapping revealed that compound 1a possessed broader non-blocker-associated aromatic regions with more localized blocker-associated contributions, whereas Talazoparib displayed more pronounced blocker-associated heterocyclic and carbonyl regions. These observations are consistent with the results obtained using QikProp. Overall, the predictions for the hERG liability of compound 1a suggest that it has a predicted profile of hERG liability that is broadly comparable to Talazoparib but with an improved margin of safety. However, these are predictions and must be validated by experimental data from in vitro assays to measure hERG activity as well as in vivo toxicity studies.

## 3. Methods and Materials

### 3.1. Artificial Intelligence-Driven Drug Design (AIDD) Employing AIDDISON^TM^ v2023

The AIDDISON^TM^ v2023 software is an AI-powered tool from EMD Millipore Corporation (Merck) that combines the power of artificial intelligence (AI) and computer-aided drug design (CADD) into a single integrated platform for virtual screening, scaffold hopping, hit identification and lead optimization [29]. AIDDISON™ v2023 uses generative methods and machine learning (ML) models trained on experimentally validated ADMET data to guide search in ultra-large chemical spaces and de novo design of “drug-like” and synthetically viable compounds. AIDDISON™ v2023 also encompasses SA-space^TM^, a synthetically accessible chemical space of 25 billion virtual compounds built on the Sigma-Aldrich^®^ catalog of molecules [29]. These compounds are readily available for purchase and have well-known robust chemical transformation rules.

### 3.2. PARP-Tailored Database Design Through AIDD and Ultra-Large Chemical Space Libraries

**Figure 15:**
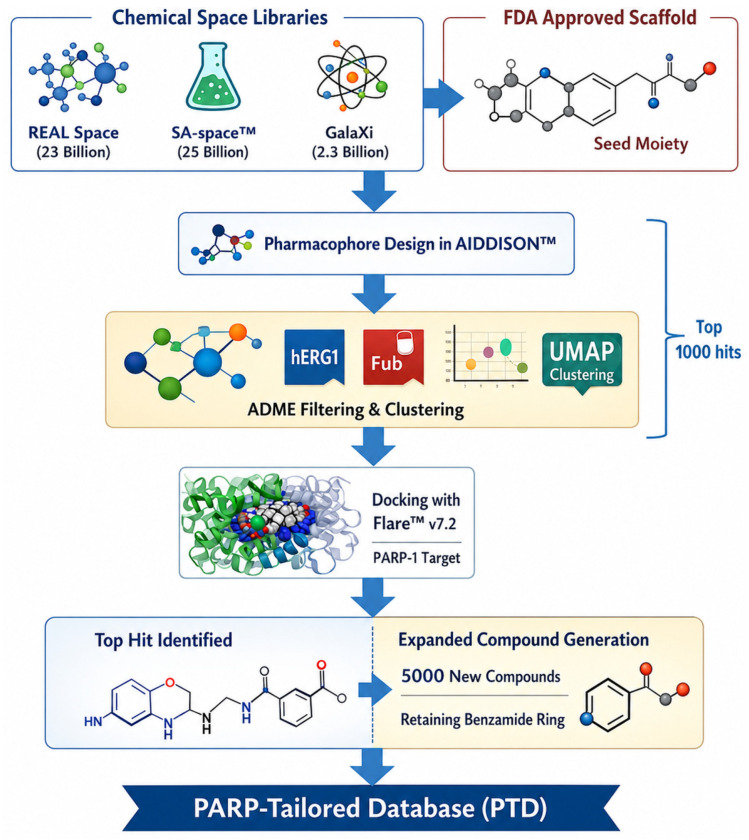
Schematic diagram of the PARP-Tailored Database design process.

In this study, the PARP-Tailored Database (PTD) was designed using the pharmacophore drug design module within AIDDISON^TM^ v2023 to create a novel set of 5000 compounds employing all three chemical space libraries (REAL space, SA-space^TM^ and GalaXi) integrated within AIDDISON^TM^ v2023 as well as the signature scaffold from the FDA- approved inhibitors as a seed moiety (Figure 15). After this process, the compounds were filtered (based on desirable properties such as hERG, Fub (fraction unbound in plasma) & the Lipinski’s rule of five) and clustered through the Uniform Manifold Approximation and Projection (UMAP)—an advanced principal component approach embedded within AIDDISON^TM^ v2023 to determine the best possible hits [29]. The top one thousand compounds were then docked using the Flare^TM^ v7.2 module, also in AIDDISON^TM^ v2023, against the PARP-1 catalytic domain on which the first hit was identified. This top-identified hit was then used to generate an additional 5000 novel compounds through pharmacophore design by retaining specified rings (e.g., benzamide) through a pharmacophoric constraint that contributes to the inhibitory activity of the binding coordination (Figure 15). This was also computed using all three chemical space libraries; that is, REAL space (23 billion), SA-space^TM^ (25 billion) and GalaXi (2.3 billion) [29]. The use of these ultra-large libraries enabled the exploration of a wider chemical space and significantly increased the novelty of the final database, whilst the pharmacophore module ensured that we obtain synthetically feasible hits (Figure 15).

### 3.3. Molecular Docking Studies

#### 3.3.1. Protein Retrieval and Preparation

The catalytic domain crystal structure of the Poly [ADP-ribose] polymerase 1 (PARP-1) was retrieved from the protein data bank—https://www.rcsb.org/ (accessed on 18 October 2025) with PDB ID: 4UND [30]. This protein was used as a receptor and prepared to a suitable state for computational calculations using the Protein Preparation Wizard in Maestro v2023-2. During this process, missing disulfide bonds, hydrogen atoms, side chains and loops were added using Prime. Water molecules beyond 3 Å of the het groups were removed and the hydrogen bonds were optimized to avoid steric clashes. Refinement of the protein structure was done through restrained minimization to an RMSD of 0.3 Ås.

#### 3.3.2. Ligand Preparation

The final ligand database was downloaded from AIDDISON^TM^ v2023, saved in .sdf format and uploaded into the Glide VSW panel and prepared using the integrated LigPrep module in Maestro v2023-2. During this ligand preparation process, all 5000 chemical compounds were desalted, and hydrogen atoms were added using Prime. The generation of various ionization states was done at a targeted pH range of 7.0 ± 2.0 using EpiK, and the stereochemical computation was set to retain specified chiralities generating up to 32 compounds per ligand. These compounds were ultimately converted to their lowest-energy-possible 3D conformation state using the OPLS4 force field and the final dataset size was expanded to 19,717 compounds.

#### 3.3.3. Grid Generation and Search Space Mapping

The receptor grids computed via the grid generation panel in Maestro were set in the center of the Talazoparib inhibitor within the protein’s active site. Thus, the outer grid orthorhombic box adopted a pre-set coordinate system of −2.60, 39.42 and 14.64 in x, y, z axis respectively whilst the inner grids were kept at 10 Å for x, y and z coordinates. These grids are critical during docking as they give a true measure of the effective search space and enable all ligands to find usual or asymmetric binding modes within the active site whilst confining their midpoints into a smaller box to save calculation time. The van der Waals radius scaling was set to 1 to soften the potential for nonpolar parts of the receptor, and the partial charge was set to 0.25.

#### 3.3.4. Molecular Docking Validation Protocol

To confirm the reliability of molecular docking for the PARP-1 protein (PDB id: 4UND), a validation study was performed using the Schrödinger suite v2023-2, on which the co-crystallized Talazoparib was redocked into its native binding site to assess the ability of the docking method in predicting the experimentally determined pose. The protein structure was prepared using the Protein Preparation Wizard [31] as described in Section 3.3.1. Additionally, the Talazoparib ligand was prepared for docking using LigPrep at pH 7.0  ±  2.0 using the OPLS4 force field to ensure the correct stereochemistry and tautomers are computed prior to docking. The receptor grid was prepared using the receptor grid generation tool with the x, y and z coordinates of the co-crystallized ligand [32]. The redocking of Talazoparib into the binding site of PARP-1 was performed using the Glide XP (Extra Precision) docking module with docking parameters identical to those used in virtual screening. The van der Waals scaling factor was set to 1.0 and the partial charge cutoff for receptor atoms was 0.25. The accuracy of docking was confirmed by calculating the RMSD between the top-ranked pose predicted by the docking protocol and the original crystallographic ligand pose. An RMSD of ≤2.00 Å was taken to be a good indicator of the pose being accurately reproduced [24].

#### 3.3.5. Structure-Based Virtual Screening Workflow

The Virtual Screening Workflow (VSW) comprised the following modules: QikProp, Lipinski’s rule of five filters, High-Throughput Virtual Screening (HTVS), Standard Precision (SP), and lastly Extra Precision (XP), which were used for screening the final PARP-Tailored Database to determine the most potent inhibitors. The QikProp module filtered the compounds based on ADMET features (absorption, distribution, metabolism, excretion and toxicity). The obtained compound list was further subjected to Lipinski’s rule of five filters. The returned compounds were then subjected to a three-step docking regime with increasing precision using the Glide module. This involved docking the compounds against PARP-1 using HTVS, SP and lastly XP. The top three complexes were further used to build a solvation system for molecular dynamics simulations.

### 3.4. Molecular Dynamics and Molecular Mechanics–Generalized Born Surface Area (MM/GBSA) Calculations

#### 3.4.1. Molecular Dynamics Simulations

The four protein–ligand complexes constituting PARP-1, the reference Talazoparib as well as the potential inhibitors—compounds 1a, 1b and 1c—were exposed to MD simulation employing the Desmond module on the Schrödinger suite v2023-2 with the OPLS4 force field as described in one of the previous studies [33]. An orthorhombic box was used to restrict both complexes, on which the TIP4P water solvation model was utilized [34]. The volume of the box was minimized to 330 913 Å^3^, thus positioning the complex at the center of the solvation box with a 10 Å buffer in the x, y, and z axis. To further mimic physiological conditions, a salt concentration of 0.15 M was added. Constant temperature (300 Kelvin) and pressure (1.01325 bar) were achieved using the Nose–Hoover thermostat [35] and Martyna–Tobias–Klein barostat [36] methods respectively through the NPT ensemble class, which ensured that the number of particles, pressure, and temperature were kept constant.

The long-range electrostatic interactions were determined during simulations employing the Particle–Mesh–Ewald method [37]. To determine the relative stability of the ligand in the receptor-binding sites, the root mean square deviation (RMSD) and root mean square fluctuation (RMSF) plots for the Poly [ADP-ribose] polymerase 1 and the top three compounds plus Talazoparib were generated using the following equations:

(1) $${RMSD}_{X}=\sqrt{\frac{1}{N}}\sum_{i=1}^{N}(r_{i}^{\prime }\left(t_{x}\right)-r_{i}(t_{ref}){)}^{2}$$

where the RMSD_X_ is referred to as the calculation for a frame x, N is the number of atoms in the atom selection; tref is the reference time (typically the first frame is used as the reference and it is regarded as time t  =  0); and r′ is the position of the selected atoms in frame x, after superimposing on the reference frame, where frame x is recorded at time t_x_. The procedure is repeated for every frame in the simulation trajectory. The RMSF was calculated using the following equation:

(2) $${RMSF}_{i}=\sqrt{\frac{1}{T}}\sum_{t=1}^{T}\left\langle (r_{i}^{\prime }\left(t\right)-\right.\left.r_{i}(t_{ref}){)}^{2}\right\rangle$$

where RMSF_i_ refers to generic residue i, T is the trajectory time over which the RMSF is calculated, tref is the reference time, ri is the position of residue i; r′ is the position of atoms in residue i after superposition on the reference, and the angle brackets indicate that the average of the square distance is taken over the selection of atoms in the residue. All simulations were monitored for 100 ns, and each had a 100 ps recording interval and generated 1000 frames. However, an additional 250 ns was also executed with a particular focus on the top ranked hit amongst the designed compounds. The results were subsequently analyzed and imagined by Simulation Interaction Diagrams and MD trajectory analysis.

#### 3.4.2. Molecular Mechanics–Generalized Born Surface Area (MM/GBSA) Calculations

To examine the stability and behavior of the protein–ligand complexes at the different simulation stages, various post-simulation analysis tasks were implemented. These included the RMSD (root mean square deviation) and RMSF (root mean square fluctuation), which were performed using the Simulation Interaction Diagram (SID) tool on the Desmond software (Schrödinger Release 2023–2). Stable segments are determined from the plateau phase shown in the RMSD trajectories. Thus, these segments of the trajectory were selected for further analysis. Flexible residues and dynamically stable protein backbone fragments of the protein–ligand complexes were confirmed from the RMSF plots. The binding affinities between the protein and the ligand are characterized from the binding free energy (ΔGbind) calculations that were implemented using the MM/GBSA (Molecular Mechanics/Generalized Born Surface Area) method [38]. The solvent molecules and counterions were removed from the protein–ligand systems prior to the energy calculation. The total binding free energy, ΔGbind, is given in the following Equation:

ΔGbind = ΔGcoulomb + ΔGcovalent + ΔGHbond + ΔGvdw + ΔGLipo + ΔGpacking + ΔGsolv(3)

According to the MM/GBSA model, the binding free energy was decomposed into thermodynamic components and molecular interaction energy items, including ΔGcoulomb (Coulombic interaction energy), ΔGcovalent (covalent bonds), ΔGH-bond (hydrogen bond correction), ΔGvdw (van der Waals interaction), ΔGLipo (nonpolar or lipophilic solvation), ΔGpacking (π-π stacking correction) and ΔGsolv (Generalized Born electrostatic solvation energy) [38]. All jobs were carried out by using the MobaXterm v25.2 ssh terminal interface by executing a bash script, and the final output data were saved in .csv format for further analysis. This provided significant data on the contributions of interaction energy items to the ligand binding affinity and stability of the protein–ligand complex.

### 3.5. Quantum Chemical Calculations

The Frontier Molecular Orbitals (FMOs) of the molecule were calculated by means of density functional theory (DFT) using M06-2X functional with def2tzvp basis set in Gaussian 16 software [39,40]. The use of polarized basis sets gives a reliable description of the electron density which further provides a premise picture for subsequent analysis of reactivity [41,42]. Molecular graphics were generated by means of GaussView 6.1.1 [43]. FMO (highest occupied molecular orbital, HOMO, and lowest unoccupied molecular orbital, LUMO) theory is one of the most quantum chemical approaches that was used to understand electronic structure, reactivity and stability of the molecules [44].

This energy gap (∆Egap) between the highest occupied molecular orbital (HOMO) and lowest unoccupied molecular orbital (LUMO) influences the stability and chemical reactivity of the molecule. The large gaps indicate higher stability and lower reactivity, whilst smaller gaps indicate higher polarizability and reactivity [45,46]. The HOMO is capable of acting as an electron donor by interacting with positively charged species and, conversely, the LUMO is capable of acting as an electron acceptor by interacting with certain species with negative charges [47,48]. The interactions between the HOMO and LUMO of a system that contains π-bonding leads to intramolecular charge transfer through the whole system and that influences the conductivity and biological activities of such molecules [49].

Additionally, the global reactivity descriptors, derived from Koopman’s theorem [50] for closed-shell systems, further characterize molecular behavior [51,52]. These descriptors include chemical hardness (η) [53], softness (δ) [54], chemical potential (Cp) [55], electronegativity (χ) [56], and electrophilicity index (ω) [57], calculated as follows:

(4) $$\eta =\frac{\left(I-A\right)}{2}$$

(5) $$\delta =\left(\frac{-1}{2\eta }\right)$$

(6) $$Cp=\frac{\left(I+A\right)}{2}$$

(7) $$\chi =\frac{\left(I-A\right)}{2}$$

(8) $$\omega =\left(\frac{{Cp}^{2}}{2\eta }\right)$$

where the ionization potential (I) and electron affinity (A) were approximated as I  =  −E_HOMO_ and A  =  −E_LUMO_, respectively [58,59]. Molecules with large energy gaps are classified as “hard,” exhibiting lower polarizability and higher stability, while those with smaller gaps are “soft,” indicating greater reactivity owing to facile electronic excitation [58]. The molecular structures of all four compounds (Talazoparib and compounds 1a–1c) were subjected to quantum optimization prior to FMO and descriptor calculations; this ensured reliable geometric and electronic representations [60]. This integrated computational approach (calculation of FMO and global reactivity descriptors) provides a comprehensive framework for predicting molecular interactions and designing compounds with tailored chemical properties, and in this study, this was executed in detail as was done in one of the recent computational studies [61]. This ensured accurate execution, as such approaches are critical in enabling the precise characterization of molecular reactivity profiles, which was significant in determining the molecular stability and reactivity of our top three lead compounds.

### 3.6. Pharmacokinetics (ADMET) Calculations

The ADMET properties for the compounds were determined using the QikProp module from the Schrödinger suite v2023-2 [27]. This was done with a special focus on the following properties: dipole, donorHB, acceptHB, QPlogPo/w, No. of metabolites, QPlogKhsa, RuleOfFive, QPlogHERG and %Human oral absorption. These parameters were compared against the recommended values which was critical in further assessing and identifying compounds with good ADMET properties. Additional toxicity assessments were performed using ProTox-3.0 (https://tox.charite.de/protox3/) [62] and Pred-hERG V5.0 (https://predherg.labmol.com.br/) web servers [63]. These tools were both accessed on the 14 May 2026.

## 4. Conclusions

This study employed an integrated computational workflow comprising artificial intelligence-driven drug design, molecular docking, molecular dynamics simulations, MM/GBSA binding free energy calculations, DFT analysis, and ADMET predictions to identify potential PARP-1 inhibitors for breast cancer therapy. Initially the docking efficiency was validated by redocking Talazoparib, a known and FDA-approved PARP-1 inhibitor, into the active site. A comparative view of the predicted docking pose with that obtained from crystallographic studies revealed an RMSD value of 0.995 Å, confirming the accuracy of the docking methodology as well as the grid and scoring parameters used. Among the evaluated compounds, 1a demonstrated the most favorable overall computational profile, including a superior docking score (−9.488 kcal/mol) and binding free energy (ΔG_bind = −67.820 kcal/mol) compared to Talazoparib (−6.778 kcal/mol and ΔG_bind = −63.734 kcal/mol, respectively), while compounds 1b and 1c also exhibited comparable predicted binding affinities and stable interaction profiles. The designed compounds consistently interacted with critical catalytic site residues such as His862, Gly863, Ser904, Tyr907 and Arg878 through hydrogen bonding, hydrophobic interactions and π–π stacking interactions, which is consistent with previously reported PARP-1 inhibitor binding modes in literature [23,25,26]. Furthermore, molecular dynamics simulations indicated stable protein–ligand complexes throughout the 100 ns and the subsequent 250 ns simulations, while DFT analysis revealed favorable electronic properties, including lower HOMO–LUMO energy gaps, reduced chemical hardness and improved softness values relative to Talazoparib, suggesting improved electronic adaptability within the PARP-1 binding pocket. In addition, the ADMET predictions on QikProp suggest acceptable drug-likeness for compounds 1a–1c and lower predicted hERG-associated cardiotoxicity risk, 1a exhibited better predicated pharmacokinetics than the designed compounds as well as Talazoparib. Further toxicity assessment employing ProTox-3.0 and Pred-hERG v5.0, showing that both compound 1a and Talazoparib have low cardiotoxicity risk, also revealed concerning clinical and hepatotoxicity issues associated with Talazoparib.

Importantly, it is worth noting that the findings presented in this study are predictive in nature and are based entirely on in silico computational approaches. While the theoretical results of the designed compounds revealed favorable binding affinities, stable interactions and favorable pharmacokinetic properties, it is worth noting that positive results of calculations may not necessarily translate into biological activity, therapeutic effects or safety in biological systems. Therefore, experimental validation by in vitro enzymatic inhibition assays, cell-based studies and in vivo experiments is required to confirm the bioactivity, pharmacological activity and therapeutic potential of the developed compounds. This work represents a good and critical starting point for future design optimizations and experimental investigations of novel PARP-1 inhibitors for the treatment of breast cancer.

## Figures and Tables

**Table 1:** Calculated docking scores.

Reference + Potential Drugs	Docking Score (XP) (Kcal/mol)	XP GScore (Kcal/mol)	Glide emodels (Kcal/mol)	Coordinating Residues (H-Bond + π-π Stacking)
Talazoparib	−6.778	−6.778	−57.728	Gly863, Tyr907
1a	−9.488	−9.715	−70.279	His862, Gly863, Ser904
1b	−9.349	−9.500	−71.371	His862, Gly863, Ser904, Arg878
1c	−9.255	−9.437	−62.141	His862, Gly863, Ser904, Arg878

Note: H-bond = hydrogen bonding, Gly = glycine, His = histidine, Ser = serine, Arg = arginine.

**Table 2:** Calculated free energy of binding ΔG (bind) and contributing energetic parameters using MD trajectories.

Ligand	ΔG_bind_	ΔG_Coulomb_	ΔG_Covalent_	ΔG_Hbond_	ΔG_Lipo_	ΔG_pack_	ΔG_Solv_	ΔG_VdW_
Talazoparib	−63.734	−15.999	1.439	−1.279	−20.548	−7.591	30.284	−50.041
1a	−67.820	−17.976	2.807	−2.541	−27.652	−4.211	26.034	−66.146
1b	−61.573	−59.375	2.973	−2.069	−13.927	−2.186	63.643	−50.632
1c	−61.329	−66.054	3.003	−2.129	−13.860	−2.315	67.968	−47.942

**Table 3:** DFT-calculated chemical reactivity indices of Talazoparib and the top 3 hits using the M06-2X level of theory with the def2tzvp basis set.

Parameters	Talazoparib	Compound		
		1a	1b	1c
E_HOMO,_ eV	−0.948	2.281	2.131	2.104
E_LUMO,_ eV	−7.606	−3.176	−3.131	−3.173
Energy gap (∆G), eV	6.659	5.456	5.262	5.277
Electron affinity (EA)	0.948	−2.281	−2.131	−2.104
Ionization potential (IP), eV	7.606	3.176	3.131	3.173
Chemical hardness (ƞ), eV	3.330	2.728	2.631	2.639
Chemical softness (δ), eV	0.300	0.367	0.381	0.379
Electronegativity (χ), eV	4.277	0.447	0.500	0.534
Chemical potential (µ), eV	−4.277	−0.447	−0.500	−0.534
Electrophilicity index (ω), eV	2.747	0.037	0.048	0.054

**Table 4:** In silico pharmacokinetic (ADMET) screening of Talazoparib and the top three compounds targeting PARP-1.

Reference + Hits	Dipole	QPlogBB	QPlogHERG	QPlogPo/w	No. of Metabolite	QPlogKhsa	Rule of Five	% Human Oral Absorption
Talazoparib	0.00	−1.278	−5.143	3.168	3	0.439	0	91.382
1a	1.270	−1.083	−4.543	2.949	4	0.074	0	82.384
1b	3.221	−1.081	−4.625	2.049	2	−0.214	0	75.220
1c	1.709	−0.585	−4.605	2.097	2	−0.205	0	75.168
Recommended Values [27,28]	1–12.5	−3–1.2	>−5 desirable <−5 poor	−2–6.5	1–8	−1.5–1.5	Max 4	>80% is high <25% is poor

NOTE: Dipole—computed dipole moment of the molecule. QPlogBB—predicted brain/blood partition coefficient. QPlogHERG—a predictive value for the potential of a molecule to block the hERG potassium channel, which is linked to cardiac toxicity. QPlogPo/w—predicted octanol/water partition coefficient. No. of Metabolite—number of likely metabolic reactions. QPlogKhsa—prediction of binding to human serum albumin. Rule of Five—number of violations of Lipinski’s rule of five. %Human Oral Absorption—predicted human oral absorption on a 0 to 100% scale. The prediction is based on a quantitative multiple linear regression model.

**Table 5:** Comparative toxicity assessment of compounds 1a–1c and Talazoparib.

Parameter	Compound 1a	Compound 1b	Compound 1c	Talazoparib
Predicted LD50 (mg/kg)	1200	883	435	500
Toxicity Class	3	4	4	4
Cardiotoxicity	Inactive (0.88)	Inactive (0.88)	Inactive (0.88)	Inactive (0.80)
Hepatotoxicity	Inactive (0.57)	Inactive (0.57)	Inactive (0.57)	Active (0.63)
Neurotoxicity	Active (0.72)	Active (0.78)	Active (0.78)	Active (0.92)
Nephrotoxicity	Inactive (0.59)	Inactive (0.59)	Inactive (0.59)	Inactive (0.58)
Carcinogenicity	Inactive (0.55)	Inactive (0.56)	Inactive (0.56)	Inactive (0.54)
Immunotoxicity	Inactive (0.78)	Inactive (0.90)	Inactive (0.80)	Inactive (0.73)
Mutagenicity	Inactive (0.59)	Inactive (0.58)	Inactive (0.58)	Inactive (0.57)
Cytotoxicity	Inactive (0.64)	Inactive (0.64)	Inactive (0.64)	Inactive (0.81)
Clinical Toxicity	Inactive (0.59)	Active (0.55)	Active (0.55)	Active (0.67)

**Table 6:** Comparative Pred-hERG v5.0 cardiotoxicity assessment of compound 1a and Talazoparib.

Parameter	Compound 1a	Talazoparib
Consensus Prediction	Non-blocker	Non-blocker
Binary Model Prediction	Non-blocker	Non-blocker
Binary Confidence (%)	69.4	82.7
Multiclass Prediction	Weak blocker	Moderate blocker
Multiclass Confidence (%)	41.5	51.2
Regression Model (pIC50)	5.267	5.374
Applicability Domain Similarity (%)	36.4	30.6
Dominant Fragment Contributions	Predominantly non-blocker aromatic scaffold with localized blocker-associated polar regions	Mixed non-blocker aromatic contributions with stronger blocker-associated heterocyclic and carbonyl regions
Predicted hERG Liability	Low	Moderate
Overall Predicted Cardiotoxicity Profile	Slightly improved	Reference compound

## Data Availability

The original contributions presented in this study are included in the article. Further inquiries can be directed to the corresponding author.

## Abbreviations

The following abbreviations are used in this manuscript.

AIDD	Artificial Intelligence Drug Design
ADMET	Administration, Distribution, Metabolism, Elimination and Toxicity
BER	Base Excision Repair
*BRCA*	Breast Cancer Gene
DFT	Density Functional Theory
DNA	Deoxyribonucleic Acid
DSB	Double-strand Break
FDA	Food and Drug Administration
FMO	Frontier Molecular Orbital
FUB	Fraction Unbound In plasma
HOMO	Highest Occupied Molecular Orbital
HRR	Homologous Recombination Repair
HTVS	High-Throughput Virtual Screening
ICT	Intramolecular Charge Transfer
LUMO	Lowest Unoccupied Molecular Orbital
MD	Molecular Dynamics
MM/GBSA	Molecular Mechanics–Generalized Borne Surface Area
PARP-1	Poly (ADP-ribose) polymerase 1
PK	Pharmacokinetics
PTD	PARP-Tailored Database
QSAR	Quantitative Structure Activity Relationship
RMSD	Root Mean Square Deviation
RMSF	Root Mean Square Fluctuation
SID	Simulation Interaction Diagram
SP	Standard Precision
SSB	Single-strand Break
UMAP	Uniform Manifold Approximation and Projection
XP	Extra Precision
